# Elasto-inertial microfluidic separation of microspheres with submicron resolution at high-throughput

**DOI:** 10.1038/s41378-023-00633-w

**Published:** 2024-01-22

**Authors:** Hyunwoo Jeon, Song Ha Lee, Jongho Shin, Kicheol Song, Nari Ahn, Jinsoo Park

**Affiliations:** 1 Department of Mechanical Engineering, Chonnam National University, 77 Yongbong-ro Buk-gu, Gwangju, 61186 Republic of Korea; 2 Analytical Engineering Team, Samsung Display Co., Ltd., 181 Samsung-ro, Tangjeong-myeon, Asan-si, Chungcheongnam-do, 31454 Republic of Korea

**Keywords:** Engineering, Physics, Other nanotechnology

## Abstract

Elasto-inertial microfluidic separation offers many advantages including high throughput and separation resolution. Even though the separation efficiency highly depends on precise control of the flow conditions, no concrete guidelines have been reported yet in elasto-inertial microfluidics. Here, we propose a dimensionless analysis for precise estimation of the microsphere behaviors across the interface of Newtonian and viscoelastic fluids. Reynolds number, modified Weissenberg number, and modified elastic number are used to investigate the balance between inertial and elastic lift forces. Based on the findings, we introduce a new dimensionless number defined as the width of the Newtonian fluid stream divided by microsphere diameter. The proposed dimensionless analysis allows us to predict whether the microspheres migrate across the co-flow interface. The theoretical estimation is found to be in good agreement with the experimental results using 2.1- and 3.2-μm-diameter polystyrene microspheres in a co-flow of water and polyethylene oxide solution. Based on the theoretical estimation, we also realize submicron separation of the microspheres with 2.1 and 2.5 μm in diameter at high throughput, high purity (>95%), and high recovery rate (>97%). The applicability of the proposed method was validated by separation of platelets from similar-sized *Escherichia coli* (*E.coli*).

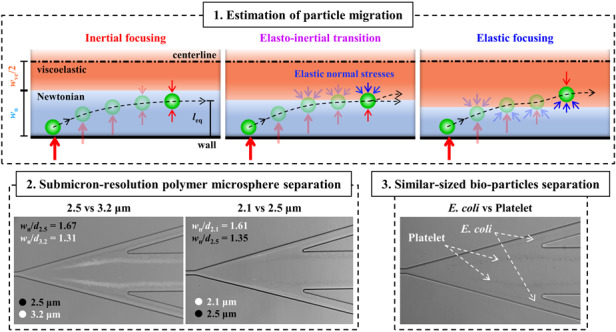

## Introduction

Sample preparation is an essential step in the overall chemical analysis process; it improves analytical results by enriching the target material or removing contaminants before analysis^1^. Particle manipulation techniques have received increasing research attention owing to their applications in sample filtration^2^, flow cytometry^3^, and separation^4^. Various microfluidic manipulation techniques with high throughput, resolution, and efficiency have been developed. In particular, regarding resolution, techniques for the precise sample separation such as red blood cells (7–8 μm)^5^, platelets (1.5–3 μm)^6^, and bacteria (1–3 μm)^7^ are highly required. However, the submicron-resolution separation of micro-objects is difficult because most of the forces moving the particles in the lateral direction are proportional to the sample size^8^.

Polymer microspheres have been widely utilized as model objects for bio-particles^9,10^. Passive manipulation techniques rely on internal hydrodynamic forces acting on suspended objects, whereas in active techniques, forces are applied to the objects from external actuation devices. The typical examples of the passive techniques include inertial flow focusing based on fluid inertial force^11,12^, pinched flow^13^ and Dean flow fractionation based on Dean drag force generated in curved pipes^14,15^, and deterministic lateral displacement (DLD) based on the streamline of flow along the structures^16^. These techniques allow simple device configuration and manipulation, offer high single-chip throughput, and enable parallelization. However, numerous parameters such as the microchannel geometry, fluid properties, and flow conditions need to be strictly tuned; otherwise, the accuracy of sample separation, efficiency, and throughput may be compromised. Conversely, in active techniques, optophoresis^17^, dielectrophoresis^18^, magnetophoresis^19^, and acoustophoresis^20^ are applied to manipulate the target samples in an on-demand manner. Despite high precision, the device configuration and parallelization are complex, and high-cost and sophisticated equipment are usually required compared to the passive approaches.

Elasto-inertial microfluidics using viscoelastic fluids can offer a breakthrough by providing increased sample manipulation performance in addition to the advantages of conventional inertial microfluidics. In a viscoelastic fluid flow, the difference in the non-uniform normal stress acting on the suspended objects generates an elastic lift force (*F*_el_), an additional force that enables the lateral migration of the object^21–23^. Micro-objects, mostly on microspheres, manipulation studies using viscoelastic fluids^24–26^ have achieved a single equilibrium position of the microspheres^9,27^, in contrast with conventional inertial microfluidics, where particles have more than two equilibrium positions^28^. Moreover, elasto-inertial microfluidic separation using a co-flow of Newtonian and viscoelastic fluids have been recently conducted to improve the sample separation performance^29–34^. The co-flow allows the wall-induced lift force (*F*_w_) and shear-gradient lift force (*F*_s_), which drive the lateral displacement of the particles suspended in the Newtonian fluid, to promote or suppress the migration of the particles owing to the generation of *F*_el_; this force can be used to increase the separation resolution. To identify the correlation between these forces under various flow conditions, dimensionless numbers can be applied. In general, in the co-flow of Newtonian and viscoelastic fluids, Reynolds number (Re)^29^, Weissenberg number (Wi)^35^, and elastic number (El = Wi/Re)^29,36^ have been previously applied for theoretical analysis. However, from the viewpoint of particle behaviors, we found that these dimensionless numbers have limitations, especially in the co-flow configuration of elasto-inertial microfluidics.

In this study, we introduced modified Wi (Wi_m_) and modified El (El_m_ = Wi_m_/Re) for investigation of the microsphere lateral migration across the interface of Newtonian and viscoelastic fluids. For thorough validation, we conduct a series of experiments with polystyrene (PS) particles with diameters of 2.1 and 3.2 μm under varying flow conditions, in which the proposed modified El is found to provide a better understanding of the suspended object behavior in the co-flow elasto-inertial microfluidics. We further introduce a new dimensionless number defined as the Newtonian fluid stream width divided by the microsphere diameter. Based on this dimensionless number, we determine three regimes of inertial focusing, elasto-inertial transition, and elastic focusing for the lateral migration of microspheres from Newtonian to viscoelastic fluids across the interface. Based on theoretical findings, we could precisely predict the microsphere behavior and thus achieve the submicron separation of the PS microspheres with diameters of 2.1 and 2.5 μm, as well as 2.5 and 3.2 μm at high throughput, high purity, and high recovery rate. The proposed elasto-inertial microfluidic separation was applied for separation of similar-sized bio-particles: platelets and *Escherichia coli* (*E. Coli*) to validate the practical applicability.

## Working mechanism

### Device configuration and elasto-inertial separation of microspheres

**Fig. 1:**
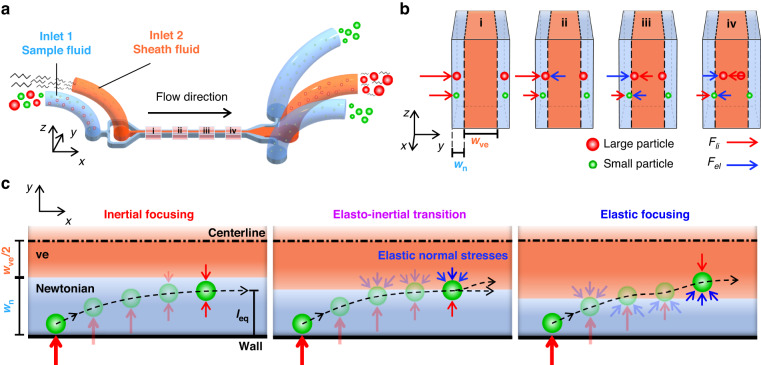
Schematic of elasto-inertial microfluidic system. **a** Schematic of a microfluidic device for size-selective microsphere separation using a co-flow of Newtonian and viscoelastic fluids. The Newtonian sample fluid (sky blue, Deionized (DI) water) with suspended particles and viscoelastic sheath fluid (orange, PEO solution) were injected by inlets 1 and 2, respectively. Large (red) and small (green) particles flow along a rectangular microchannel formed by the three-layered co-flow. The small particles in the Newtonian fluid medium and the large particles in the viscoelastic fluid medium exit through the side outlets and a center outlet, respectively. **b** Schematic of the microsphere separation mechanism following the midstream region i–iv in Fig. 1a. **c** *xy*-plane view of the microsphere migration mechanism and equilibrium positions according to the location of Newtonian/viscoelastic fluid interface. The microsphere migration is categorized into three regimes: inertial focusing, elasto-inertial transition, and elastic focusing

Figure 1a schematizes the microfluidic device, composed of two inlet ports and three outlet ports, for the elasto-inertial separation of microspheres. A sample fluid (DI water solution with suspended PS microspheres) and a sheath fluid (dilute polyethylene oxide (PEO) solution) were introduced through inlets 1 and 2, respectively. The sample fluid flow, represented in sky blue in Fig. 1, was bifurcated at the upstream to sandwich the sheath fluid in the center, presented in orange, at midstream to form a three-layered co-flow of the sample/sheath/sample fluids. Figure 1b represents the sequential transfer of the microspheres along the microchannel in the regions i–iv marked in Fig. 1a. The microspheres suspended in the Newtonian fluid are initially aligned along the two side-walls of the microchannel by the viscoelastic sheath fluid flow (Fig. 1b (i)). The microspheres near the walls experience a wall-induced lift force (*F*_w_) owing to the increased pressure between the wall and the microspheres and consequently migrate away from the wall toward the Newtonian/viscoelastic fluid interface. Because *F*_w_ is proportional to the microsphere diameter (*d*) such that *F*_w_ ∝*d*^6^, the larger microspheres experience *F*_w_ with greater magnitude and thus migrate faster away from the wall compared with the smaller microspheres (Fig. 1b (ii))^37^. The larger microspheres positioned across the Newtonian/viscoelastic fluid interface begin to experience an elastic lift force (*F*_el_) induced by the elastic effect of the viscoelastic fluid. The elastic lift force can be expressed as *F*_el_ = *C*_el_*d*^3^∇*N*_1_, where *C*_el_ is the elastic lift coefficient, *N*_1_ = *σ*_*xx*_ – σ_*yy*_ is the first normal stress difference, and *σ*_*xx*_ and *σ*_*yy*_ are the stress tensor of the normal and transverse directions in the fluid flow, respectively. The first normal stress difference can be expressed as *N*_1_ = 2*μ*_p_*λγ̇*^2^, where *μ*_p_ is the polymeric contribution to the solution viscosity, *λ* is the relaxation time, and *γ̇* is the average fluid shear rate obtained using the Oldroyd-B constitutive model^38^. *F*_el_ acts toward the side wall when the majority of the microspheres are located in the Newtonian fluid (Fig. 1b (ii)), whereas it acts toward the microchannel center when the majority of the microspheres are located in the viscoelastic fluid (Fig. 1b (iii))^33^. The smaller microspheres approach the Newtonian/viscoelastic fluid interface after the larger microspheres transfer from the Newtonian to the viscoelastic fluid. The microspheres also experience a shear-gradient lift force (*F*_s_∝*d*^3^), which acts toward the side wall, owing to the relative velocity of the microspheres moving along with the fluid flow^37^. The larger microspheres suspended in the viscoelastic fluid migrate further to two equilibrium positions, which are determined by *F*_s_ and *F*_el_ acting toward the side wall and the microchannel center, respectively (Fig. 1b (iv)). In contrast, the smaller microspheres, more than half of which are positioned in the Newtonian fluid, are translocated to their equilibrium positions determined by *F*_w_ and *F*_el_ acting toward the microchannel center and the side wall, respectively. Unlike the larger microspheres, the two competing forces acting on the smaller microspheres form the two equilibrium positions near both side walls in the Newtonian fluid (Fig. 1b (iv)). The following section presents a detailed explanation on the equilibrium positions of the microspheres. Thus, the larger microspheres change their medium from the Newtonian to the viscoelastic fluid, whereas the smaller microspheres remain in the Newtonian fluid. At downstream expansion, the microchannel is connected to the tri-furcation with three outlet ports; thus, the larger and smaller microspheres can be separately collected through the center outlet and the two side outlets, respectively.

### Transfer of microspheres across interface of Newtonian and viscoelastic fluids

The equilibrium positions of the microspheres are determined by the competing forces induced by both the inertial and elastic effects of the Newtonian and viscoelastic fluids. In the proposed size-based elasto-inertial separation method, we found that the relative location of the co-flow interface with reference to the microsphere equilibrium position plays a significant role. In the co-flowing configuration, the location of the co-flow interface can be determined by the volumetric flow rates of the Newtonian and viscoelastic fluids (*Q*_n_ and *Q*_ve_, respectively). Figure 1c shows a half of the microchannel with respect to the centerline in the *xy*-plane. With the increasing flow rate ratio of *α* = *Q*_ve_/*Q*_n_, the half-width of the viscoelastic sheath flow (*w*_ve_/2) increases while the width of the one-side Newtonian sample flow (*w*_n_) decreases. Depending on the relative location of the Newtonian/viscoelastic fluid interface and the microsphere equilibrium position, we categorized the microsphere migration phenomenon into three regimes: inertial focusing, elasto-inertial transition, and elastic focusing. In the inertial focusing regime with relatively low *α* conditions, *w*_n_ is sufficiently large compared with the microsphere size. The equilibrium position of the microspheres is determined by the two counteracting inertial effects of *F*_w_ and *F*_s_. The combined contributions of these two forces originating from the Newtonian fluid flow can be characterized by the total inertial lift force expressed as *F*_li_ = *C*_li_*ρU*^2^*d*^4^/*D*_h_^2^, where *C*_li_ is the inertial lift coefficient, *ρ* is the fluid density, *U* is the maximum fluid velocity, and *D*_h_ is the hydraulic diameter^39^. In other words, the distance (*l*_eq_) between the equilibrium position and the side wall is smaller than *w*_n_ (*l*_eq_ $<$ *w*_n_). Although the microspheres, initially located close to the wall, migrate away from the wall owing to *F*_w_, they cannot reach the co-flow interface and therefore remain in the Newtonian fluid in the inertial focusing regime.

In the elasto-inertial transition regime with moderate *α* conditions, the microspheres could reach the co-flow interface and thus experience both the inertial and elastic forces. As the center of the microsphere is located in the Newtonian fluid, *F*_w_ pushes the microspheres away from the wall and toward the microchannel center, whereas the elastic lift force acts in the opposite direction. The counteracting forces with comparable magnitudes (*l*_eq_ $\cong$ *w*_n_) result in the transition between the inertial and elastic focusing regimes. With increasing *α*, the Newtonian fluid width decreases down to the scale of the microspheres, resulting in *l*_eq_ > *w*_n_. In the elastic focusing regime, the microspheres initially aligned near the wall can easily reach the co-flow interface, and their center locations transfer from the Newtonian to the viscoelastic fluid. Thus, the direction of the elastic compressive stress is inverted, as illustrated in Fig. 1c. The microspheres at the co-flow interface in the elastic focusing regime experience *F*_el_, whose magnitude is greater than that of *F*_s_. Therefore, the combined inertial and elastic effects cause the translocation of the microspheres from the Newtonian to the viscoelastic fluid.

## Results and discussion

### Investigation of the Newtonian/Viscoelastic fluid interface

**Fig. 2:**
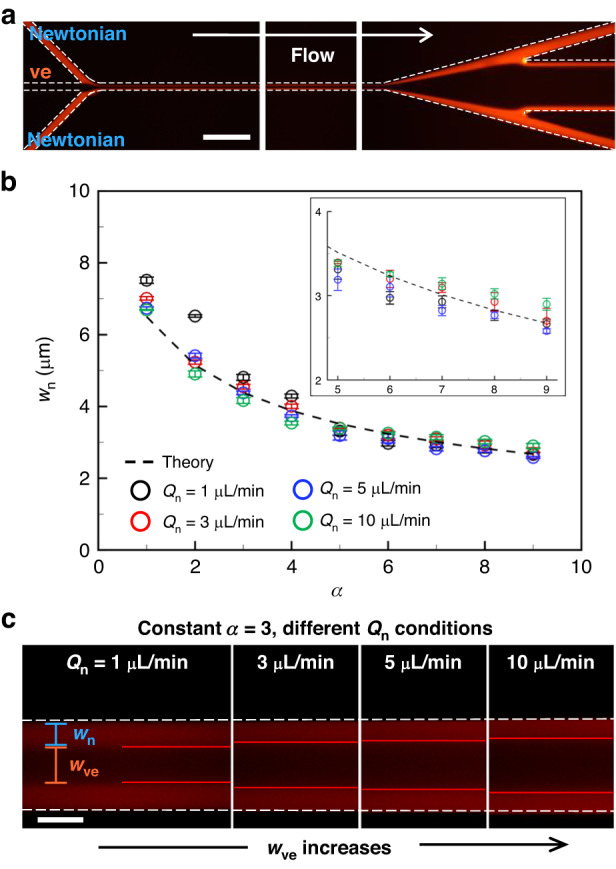
Investigation of Newtonian and vicoelastic fluids interface using the microchannel with *w* = 20 μm and *h* = 50 μm **a** Visualization of the Newtonian/viscoelastic fluid interface at a flow rate ratio of *α* = 3 (*Q*_n_ = 5 μL min^−1^, *Q*_ve_ = 15 μL min^−1^). Scale bar = 100 μm. **b** Newtonian fluid width *w*_n_ for each *Q*_n_ as a function of *α* = 1–9. The dashed line represents the theoretical prediction of *w*_n_ in a fully developed two-dimensional Poiseuille flow. The inset describes the specific information of *w*_n_ according to *α* = 5–9. **c** Variation of fluid inerface according to different *Q*_n_ = 1–10 μL min^−1^ in constant *α* = 3. Scale bar = 10 μm

As explained earlier, the translocation of microspheres across the Newtonian/viscoelastic fluid interface is governed by their equilibrium positions with reference to the co-flow interface. Thus, we investigated the interface location under varying volumetric flow rate conditions of the Newtonian and viscoelastic fluids using a microchannel with channel width *w* = 20 μm and channel height *h* = 50 μm. Figure 2a shows the stacked fluorescent microscopic images of the microchannel’s upstream, midstream, and downstream trifurcation connected to three separate outlets. We used the 300-nm red fluorescent PS particle solutions in DI water (Newtonian); no fluorescein was present in the viscoelastic fluid for identifying the location of the Newtonian/viscoelastic fluid interface. The flow rate ratio of the viscoelastic fluid to the Newtonian fluid was defined as *α*. Figure 2b shows the Newtonian fluid width (*w*_*n*_), which indicates the interface location, as a function of the flow rate ratio *α* = *Q*_ve_/*Q*_n_ under varying *Q*_ve_. The dashed line indicates the theoretical estimation of the co-flow interface location that was obtained in the following procedure. First, the streamwise velocity field *u*(*y*, *z*) was calculated by solving the conservation equations for the Newtonian fluid and assuming a Poiseuille flow in the rectangular channel cross-section in the *x*-direction. Second, the definite integral of *u*(*y*, *z*), having upper and lower limits related to the Newtonian fluid width, was calculated for each flow rate ratio^31^. The theoretical value of *w*_n_, obtained by solving the conservation equation for the Newtonian fluid excluding the elastic effect, gradually decreased as *α* increased from 1 to 9 without being influenced by the varying flow rate conditions.

Interestingly, in contrast with the theoretical estimation, the experimentally measured fluid width was found to change even with a fixed *α* in varying flow rate conditions. In Fig. 2b, at *α* = 1–4, the maximum and minimum values of *w*_n_ that decreased with increases in *Q*_n_, occurred at *Q*_n_ = 1 and 10 μL min^−1^, respectively. The development of a fluid interface at *Q*_n_ = 1 μL min^−1^, which appeared at *α* = 1–4, formed away from the microchannel wall compared with *Q*_n_ = 3–10 μL min^−1^. We estimated that the low volumetric flow rates of *Q*_n_ and *Q*_ve_ with the low Re did not stably form the co-flow. These differences in the interface position of co-flow may have been caused by the lack to push the Newtonian fluid to the two side walls due to the low inertial effect of *Q*_ve_. Therefore, the higher values of *w*_n_, under experimental conditions of *Q*_n_ = 1 μL min^−1^ and *α* = 1–4, were measured differently from the theoretical values.

Figure 2c shows the various interface locations with constant *α* = 3 when the total flow rate *Q*_total_ was 4, 12, 20, and 40 μL min^−1^. However, when *α* increased above 5, *Q*_n_ = 10 μL min^−1^, which showed the minimum value of *w*_n_ in the *α* = 1–4 range, had the widest *w*_n_ compared with *Q*_n_ = 1–5 μL min^−1^ in the inset of Fig. 2b. Under experimental conditions of *Q*_n_ = 1 μL min^−1^, as *α* increased, a sufficient flow rate and inertial effect of the viscoelastic fluid were formed to push the Newtonian fluid to the two side walls, and *w*_n_ value was similar to that obtained theoretically. At *Q*_n_ = 10 μL min^−1^, the width variation of the Newtonian fluid was limited even when *Q*_ve_ increased owing to the high inertial effect of the Newtonian fluid; as *α* increased, the change in the slope was less than that under other *Q*_n_ conditions. Therefore, at *α* = 5, the *w*_n_ reversal phenomenon of *Q*_n_ = 1 and 10 μL min^−1^ was observed.

### Modified dimensionless numbers

The parallel streams of the Newtonian and viscoelastic fluids can be characterized by three dimensionless numbers: Reynolds (Re), Weissenberg (Wi), and elastic (El)^29^. However, the previous analysis based on these dimensionless numbers offers limited information of the inertial and elastic effects in the co-flow configuration, as will be delineated later. In this regard, we introduced modified Wi and modified El in the present study. In the proposed elasto-inertial microfluidic separation method, the microspheres suspended in the Newtonian fluid laterally migrate toward the co-flow interface owing to the inertial effect, which can be characterized by Re = *ρUD*_h_/*μ*. With increasing Re, the inertial effect on the microspheres is enhanced, resulting in increased lateral migration velocity away from the wall and toward the co-flow interface. In this study, we used a dilute polymer solution (overlapping concentration c^*^ = 1877 ppm)—100 ppm PEO (*M*_w_ = 600 kDa) solution, which can be categorized as a Boger fluid^38^—as a viscoelastic fluid. The viscosity of this solution over a wide range of shear rates remains constant. The density and viscosity of the dilute PEO solution (*ρ*_ve_ = 998.3 kg m^−3^ and *μ*_ve_ = 1.041 mPa∙s) were similar to those of the Newtonian DI water (*ρ*_n_ = 998.2 kg m^−3^ and *μ*_n_ = 1.001 mPa∙s). In previous studies^29,31–33^, Re was calculated only using viscoelastic fluid properties such that Re = 2*ρ*_ve_*Q*_total_/*μ*_ve_(*w* + *h*), where *ρ*_ve_ is the viscoelastic fluid density, *Q*_total_ is the total flow rate, *μ*_ve_ is the viscoelastic fluid viscosity. Although the inertial effects are induced by both the Newtonian and viscoelastic fluids, Re is defined only for the fluid property of the viscoelastic fluid. However, we adopted the same definition for Re because (i) the properties of both fluids considered in this study were similar and (ii) the flow rate of the viscoelastic fluid was much higher than that of the Newtonian fluid in our experimental conditions for separation.

**Fig. 3:**
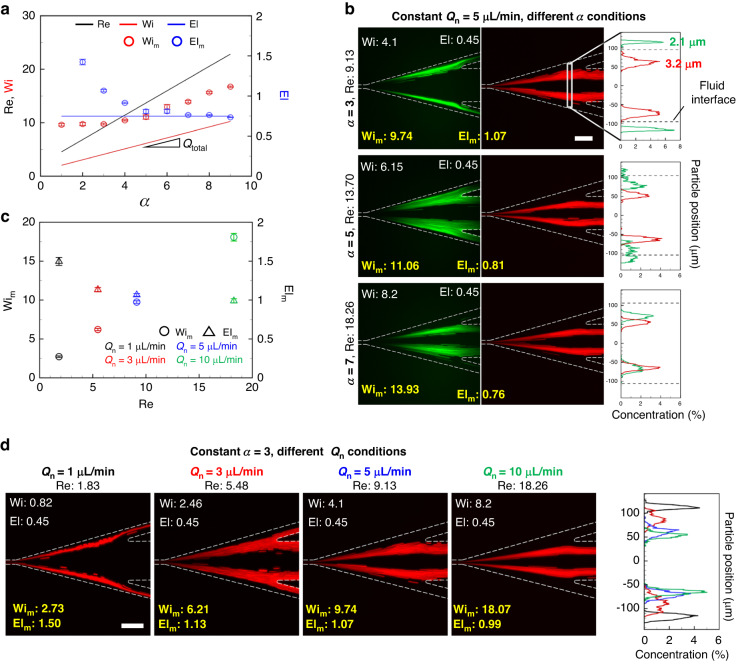
Dimensionless analysis according to microsphere trajectories in the microchannel with *w* = 20 μm and *h* = 50 μm. **a** Dimensionless values of Re, Wi, El, Wi_m_, and El_m_ according to various *α* conditions in *Q*_n_ = 5 μL min^−1^. The lines represent conventional dimensionless numbers in Newtonian and viscoelastic fluid co-flow, and the dots indicate nonlinear changes as a modified dimensionless number. **b** Fluorescence trajectories of 2.1 μm (green) and 3.2 μm (red) microspheres in *Q*_n_ = 5 μL min^−1^ and under various *α* conditions. The graphs indicate the particle positions measured 520 μm away from the expansion channel. Scale bar = 100 μm. **c** Characterization of elasto-inertial flow on a diagram with Re, Wi_m_, and El_m_ under identical *α* and various *Q*_n_ = 1–10 μL min^−1^ conditions. **d** Fluorescence trajectories of 3.2 μm (red) microspheres under identical *α* and *Q*_n_ = 1 μL min^−1^ (black), 3 μL min^−1^ (red), 5 μL min^−1^ (blue), and 10 μL min^−1^ (green) conditions. Scale bar = 100 μm

Wi measures the ratio of the elastic effects to the viscous effects of fluid flows; it is widely used to account for the elastic effect induced by viscoelastic fluids. With increasing Wi, the significance of the elastic effect increases, causing the microspheres suspended in the viscoelastic fluid to congregate in the microchannel center owing to the increased elastic lift force. Previous studies on the co-flow of Newtonian and viscoelastic fluids^29,31–33^ have defined Wi as Wi = *λγ̇* = 2*λQ*_total_/*hw*^2^, where *λ* is the relaxation time as an inherent property of viscoelastic fluids. In this definition, the total flow rate was used with the geometric dimensions of the entire microchannel cross-section (width and height). In other words, Wi was calculated using the properties and geometric characteristics of both Newtonian and viscoelastic fluids. However, in this study, we propose a modified Weissenberg number (Wi_m_) defined as Wi_m_ = 2*λQ*_ve_/*hw*_ve_^2^ by only considering the viscoelastic fluid flow. As demonstrated by the experimental results in Fig. 3, the proposed Wi_m_ better accounts for the elastic effect acting on the microspheres in a co-flow of Newtonian and viscoelastic fluids. This is attributable to the fact that the relaxation time for Newtonian fluids is zero and that the elastic effect is caused only by viscoelastic fluids and not by Newtonian fluids.

In the elasto-inertial microfluidics system, comparing the inertial and elastic effects is important to understand how the behavior of microparticles. El (Wi/Re), defined as the ratio of the elastic effect to the inertial effect, is effective in explaining the dominant effect of *F*_li_ and *F*_el_ within the channel. Previous studies^29,31–33^ have considered El to be unaffected by the changes in the flow rate of Newtonian and viscoelastic fluids because both Re and Wi are functions of *Q*_total_ and act only as functions of the viscoelastic fluid properties *μ*_ve_, *ρ*_ve_, and *λ*. This dimensionless number provides insufficient information about the lateral migration of microparticles under varying co-flow conditions and constant viscoelastic fluid properties. We propose a modified elastic number (El_m_) to describe the behavior of each particle under various flow conditions in the same PEO solution. This modified elastic number (El_m_ = Wi_m_/Re) includes not only the elastic effect according to the properties of viscoelastic fluids but also the characteristics for changing flow conditions as a function of *Q*_ve_, *Q*_total_, and *w*_ve_^2^.

1 $${{\rm{El}}}_{{\rm{m}}}=\frac{{\rm{Elastic\; effect}}}{{\rm{Inertial\; effect}}}=\frac{{{\rm{Wi}}}_{{\rm{m}}}}{\mathrm{Re}}=\frac{\lambda {\mu }_{{\rm{ve}}}}{{\rho }_{{\rm{ve}}}}=\frac{{Q}_{{\rm{ve}}}}{{Q}_{{\rm{total}}}}\frac{\left(w+h\right)}{{w}_{{\rm{ve}}}^{2}h}$$

The location of particles can be explained by comparing the inertial and elastic effects that change according to varying co-flow conditions. The dimensionless numbers presented in this study provided more detailed explanations for the behavior of particles suspended in both Newtonian and viscoelastic fluids compared with the conventional dimensionless numbers.

Figure 3a shows the difference between the conventional and modified dimensionless number under varying flow rate ratios and fixed *Q*_n_ = 5 μL min^−1^. With the increase in the volumetric flow rate of a viscoelastic fluid, the Re and Wi used in previous studies linearly increased because they are dependent on *Q*_total_. This linearity did not lead to a change in El (0.45, 100 ppm PEO–water solution) under all flow conditions. The consistent El led to difficulties in explaining the systematic analysis of the inertial and elastic effects and the migration of particles at the center of the microchannel with increasing *α*, as shown in Fig. 3b. In contrast to the conventional dimensionless number, El_m_, which is inversely proportional to the square of *w*_ve_, increased with a relatively low slope in *α* = 1–4 owing to the rapid increase in *w*_ve_. In the high *α* = 5–9, because the viscoelastic fluid flow rate is more dominant than *w*_ve_, a relatively rapid rise of Wi_m_ was observed with the increase of *α*. As *α* increased, the non-linearity of Wi_m_ led to a gradual decrease in El_m_, indicating that the fluid flow dominated the inertial effect more than the elastic effect.

The improved analysis of particle trajectories using the modified dimensionless numbers well explained the overall behaviors of the microspheres. The decrease in El_m_ with increasing *α* according to flow conditions well explained the variations in the particle trajectories, as shown in Fig. 3b. The 2.1 (green) and 3.2 μm (red) PS microspheres moved toward the center of the microchannel when *α* increased under the same Newtonian fluid flow condition *Q*_n_ = 5 μL min^−1^. The 2.1-μm particles remained in the Newtonian fluid at *α* = 3; however, the 3.2-μm particles moved across the fluid interface toward the viscoelastic fluid at *α* = 5 and 7. The migration of microspheres across the fluid interface indicates that *F*_w_ in the direction of the microchannel centerline overcomes *F*_el_ in the direction of the microchannel wall. The constant value of El = 0.45, which is not a function of fluid flow, provides a limited explanation of the inertial and elastic effects. However, El_m_ decreases from 1.07 to 0.76 as *α* increases, explaining how the 2.1-μm-diameter particles overcome the elastic lift force and move in the direction of the viscoelastic fluid flow owing to the more dominant effect of *F*_w_. The modified dimensionless numbers adequately represented not only the microsphere migration from the Newtonian fluid to the viscoelastic fluid but also the behavior of suspended microspheres in the viscoelastic fluid after passing through the interface. In Fig. 3b, the 3.2-μm red fluorescent microspheres were already suspended in the viscoelastic fluid at *α* = 3. As *α* increased, the concentration of microsphere equilibrium positions within the viscoelastic fluid intensified. This phenomenon, wherein microspheres gradually congregate in the viscoelastic fluid, is attributable to the increases in *F*_s_ and *F*_el_ in viscoelastic fluid. The increase in *Q*_ve_ strengthened the lift forces by increasing the fluid velocity and the gradient of shear rate in the Poiseuille flow, leading to the simultaneous increase in Re (from 9.13 to 18.26) and Wi_m_ (from 9.74 to 13.93).

Figure 3c illustrates the relation between Re and the modified dimensionless numbers at the same *α* = 3 under different *Q*_n_ conditions. In contrast to Re, which is only a function of the *Q*_total_ in Fig. 3c, the modified dimensionless numbers Wi_m_ and El_m_ have functional relation with fluid interface changes. At *α* = 3 in Fig. 2b, c, *w*_n_—which decreases with increase in *Q*_n_—causes nonlinear changes in Wi_m_ and El_m_ as Re increases. This nonlinearity well explains the trajectories of the microspheres when particles cross the interfaces and focus their equilibrium positions in the viscoelastic fluid. The 3.2-μm microspheres in Fig. 3d migrate toward the center of the microchannel as El_m_ decreases with increase in *Q*_n_, thus indicating the augmentation of the dominance of the inertial effect over the elastic effect. El_m_ decreases from 1.5 to 0.99, suggesting that sufficient wall-induced lift force is applied for the 3.2-μm particles to migrate in the direction of the viscoelastic fluid. The analysis of microsphere trajectories using modified dimensionless numbers overcomes the limitations of the conventional linear analysis of Wi and El and enables the overall analysis of particle trajectories and co-flow phenomena.

### New dimensionless analysis for the prediction of particle trajectories

**Fig. 4:**
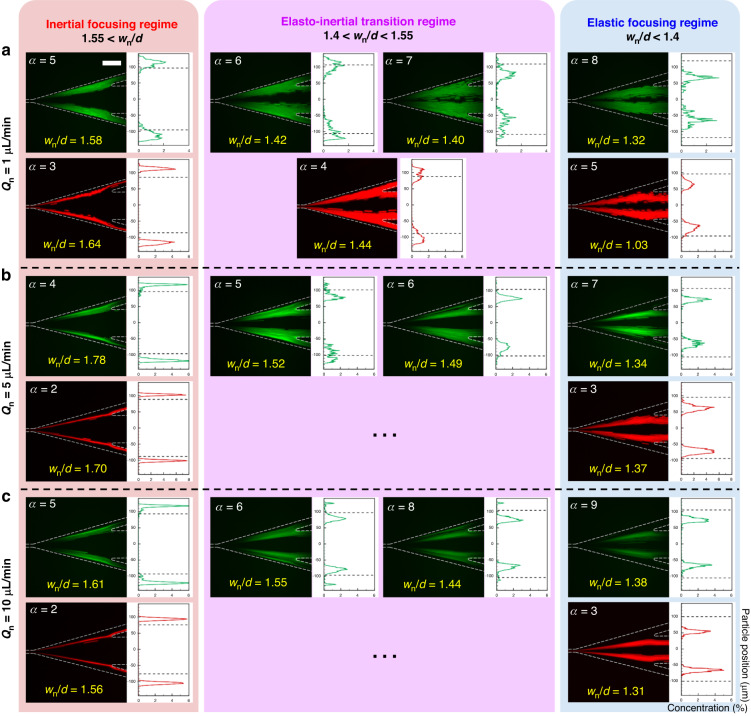
Microsphere equilibrium positions according to relationship between particle size and Newtonian fluid width in the microchannel with *w* = 20 μm and *h* = 50 μm. The three regimes are divided into inertial focusing (red, 1.55 < *w*_n_/*d*), elasto-inertial focusing (purple, 1.4 < *w*_n_/*d* < 1.55), and elastic focusing regime (sky blue, *w*_n_/*d* < 1.4). Microspheres have different equilibrium positions under varying conditions of flow rate ratio according to Newtonian fluid flow rates **a** *Q*_n_ = 1 μL min^−1^, **b** *Q*_n_ = 5 μL min^−1^, and **c** *Q*_n_ = 10 μL min^−1^ based on the experimental *w*_n_ values in Fig. 2c. Scale bar = 100 μm

Re, Wi_m_, and El_m_ adequately represented the relative difference between the inertial effect and elastic effect described by *F*_li_ and *F*_el_, respectively, and the lateral migration of the microspheres in the co-flow of Newtonian and viscoelastic fluids. However, because the modified dimensionless numbers were not functions of particle size, their abilities to describe the exact position of each particle in the co-flow were limited. In this study, we introduce a new dimensionless number *w*_n_/*d* that can estimate the three regimes (inertial focusing, elasto-inertial transition, and elastic focusing regime) according to the positions of the microspheres. The migration of the microspheres in the viscoelastic fluid was closely related to the Newtonian fluid width, and the equilibrium positions of the particles could accordingly be specified in high aspect ratio microchannel. The new dimensionless number *w*_n_/*d* is the ratio of the surface area occupied by a particle to the Newtonian fluid width. The inertial lift force that promotes the migration of a particle in the lateral direction is a function of the particle’s potion in the channel^37^. In the case of the fluid interface formed away from the microchannel wall, the particles moved in the direction of the interface owing to *F*_w_, which decreases as the particles move away from the channel wall. The increase in pressure between the microsphere and the microchannel wall became insignificant, and even if the microsphere reached the interface, *F*_w_ was not sufficient to overcome *F*_el_, as shown in Fig. 1c. We experimentally confirmed that the particles were under the inertial focusing regime, wherein the equilibrium positions were in the Newtonian fluid when *w*_n_ was 1.55 times or more compared with the particle size, as shown in Figs. 4 and S1 of Supporting information. As the fluid interface approached the microchannel wall, the microspheres adopted the elasto-inertial transition regime (1.4 < *w*_n_/*d* < 1.55), where they coexist in the Newtonian and viscoelastic fluids. In the elastic focusing regime (*w*_n_/*d* < 1.4), where the microspheres had their equilibrium positions in the viscoelastic fluid, the migration of microspheres in the Newtonian fluid under the dominant influence of *F*_w_ was promoted in the direction of the viscoelastic fluid.

The trajectories of specific microspheres had a close relationship with the particle size and Newtonian fluid width. The microsphere trajectories under all experimental conditions in this study could be determined by the numerical value of *w*_n_/*d*. Figure 4 shows microspheres experiencing the inertial focusing regime (red), in which the particles remained in the Newtonian fluid, as *w*_n_/*d* > 1.55. The increase in the flow rate of the viscoelastic fluid while maintaining constant Newtonian fluid flow rate led to a decrease in *w*_n_/*d*, which in turn caused the microspheres to enter the elasto-inertial transition regime (purple) and the elastic focusing regime (sky blue). The 2.1-μm microspheres entered the elastic focusing regime when the flow conditions were *Q*_n_ = 1 μL min^−1^ and *α* = 8, as shown in Fig. 4a. At the same Newtonian fluid flow rate *Q*_n_ = 1 μL min^−1^, the decrease in the Newtonian fluid width owing to the increasing flow rate of the viscoelastic fluid caused *w*_n_/*d* to decline from 1.58 to 1.32. At different Newtonian fluid flow rates, *Q*_n_ = 5 and 10 μL min^−1^, the entry of the 2.1-μm microspheres into the elastic focusing regime indicates *α* = 7 and 9, respectively, as shown in Fig. 4b, c. The Newtonian fluid width at *Q*_n_ = 5 μL min^−1^ was gradually reduced to bring the *w*_n_/*d* value below 1.4 at the lowest *α* condition, whereas at *Q*_n_ = 10 μL min^−1^, the microspheres entered the elastic focusing regime at the highest *α* condition because the high inertial effect of the Newtonian fluid delayed its width reduction even with increased *Q*_ve_. In the regime of *α* = 1–4, the Newtonian fluid width of *Q*_n_ = 1 μL min^−1^ was larger than that of *Q*_n_ = 5 and 10 μL min^−1^ (Fig. 2c); the relatively high *w*_n_/*d* value that was measured indicates that the 3.2-μm microspheres at *α* = 5 entered the elastic focusing regime (Fig. 4a). In the *α* conditions in this study, the elasto-inertial transition regime of 3.2-μm microspheres at *Q*_n_ = 5 and 10 μL min^−1^ is not observed owing to the rapid decrease in *w*_n_/*d*. The new dimensionless number *w*_n_/*d* can be used to specify the position of particles under all experimental conditions and effectively explains the correlation between the particle and the Newtonian fluid width.

Additionally, for comparison with the results of *w* = 20 μm in Fig. 4, we investigated the trajectories of 3.2-μm microspheres under varying channel width while the microchannel height was fixed as *h* = 50 μm (Fig. S2, Supporting Information). The microchannel width was carefully changed to ensure the channel cross-section had a high aspect-ratio rectangular shape to ensure that the migration force was dominant in the *y*-direction. The experimental results show that each trajectory regime was clearly observed, as in the case of *w* = 20 μm in Fig. 4. However, as in Fig. S2c of Supporting Information, when the aspect ratio approached to 1 (close to square cross-section) with *w* = 40 μm and *h* = 50 μm, the *w*_n_/*d* values between the three regimes have been found to slightly increase. These results could be due to decreasing *F*_s_ that suppressed the migration of the particles toward the co-flow interface, as in Fig. 1c. In the high aspect microchannel, *F*_s_ was sufficient in the *y*-direction (due to the steep velocity profile in the *y*-direction) anywhere away from the walls, but relatively weak in the *z*-direction^37,40^. In the square-like microchannel (*w* = 40 μm and *h* = 50 μm), the relatively blunt velocity profile in the *y*-direction reduced *F*_s_, and thus the microspheres moved toward the viscoelastic fluid at higher *w*_n_/*d*. However, In the case of scaling up the main-microchannel to have an aspect ratio of 2.5, the experimental results of 6.02-μm microspheres was found to be valid in our dimensionless analysis to estimate the microsphere migration behavior (Fig. S3, Supporting Information). The concentration of viscoelastic fluid can be an important variable determining of particle behaviors. It has been reported that with increasing PEO concentration, an increase in the relaxation time results in an increase elastic lift force by the viscoelastic fluid^32,33^. In this regard, we expect that the *w*_*n*_/*d* value may be decreased with increasing PEO concentration due to the enhanced elastic effect. However, we have excluded the investigation of the PEO concentration effect to focus on the effects of the relative dimensions (*w*_*n*_/*d*) and flow rate ratio (*α*) in elasto-inertial microfluidics. Instead, we plan to continue our research to fully elucidate the elasto-inertial microsphere migration by including the PEO concentration effect in a follow-up study.

**Fig. 5:**
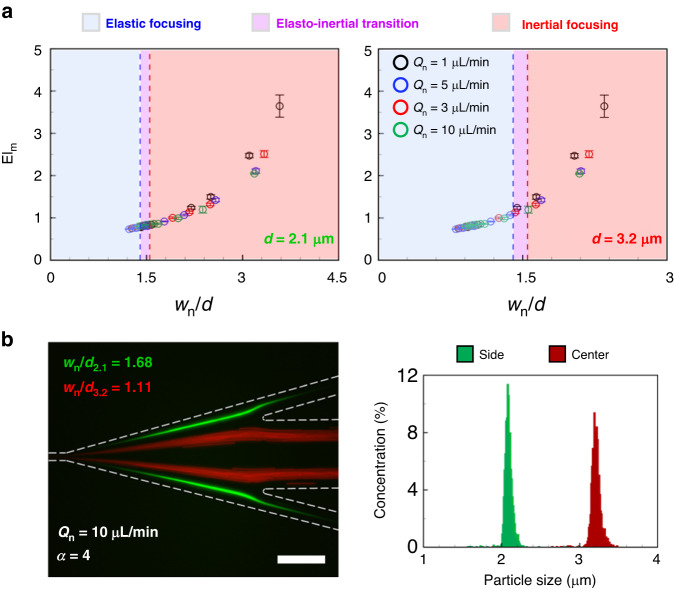
2.1 and 3.2 μm microspheres separation based on dimensionless analysis. **a** Flow analysis using the new dimensionless number and modified elastic number in the various experimental conditions of in this study. Depending on the size of each microsphere, the equilibrium positions of the microspheres are divided into three regimes based on the threshold values of *w*_n_/*d*. **b** The separation fluorescent image of 2.1 and 3.2 μm microspheres using the new dimensionless number for analysis. The size distributions indicate the collected microspheres at each side outlet (green) and the center outlet (red). Scale bar = 100 μm

All the experimental conditions in the microchannel with *w* = 20 and *h* = 50 μm are expressed using Re and Wi_m_; the correlation between the flows was difficult to obtain, and the transfer of information about particle trajectories had limitations (Fig. S4, Supporting Information). Figure 5a presents all the experimental conditions under which *w*_n_/*d* and El_m_ can be applied. Although different flow phenomena occurred under each experimental condition, which are analyzed from the particle trajectory viewpoint, the figure signifies which experimental conditions could be analyzed as the same flow phenomenon utilizing *w*_n_/*d* and El_m_. In Fig. 5a, an optimal separation condition for microspheres could be predicted simply by selecting the inertial focusing and elastic focusing regimes for 2.1 and 3.2 μm microspheres, respectively (see Video S1, Supporting Information). Our analysis allowed the convenient verification of the maximum Newtonian fluid flow rate (*Q*_n_ = 10 μL min^−1^) in terms of the microfluidic device’s throughput. Microsphere separation using the dimensionless numbers was achieved with high efficiency: 99.87% and 99.64% purity of 2.1 and 3.2 μm microspheres, respectively (Fig. 5b). The new dimensionless number *w*_n_/*d* successfully predicted the behavior of particles for complex flow phenomena occurring in the co-flow of Newtonian and viscoelastic fluids; it could also assist in selecting the parameters (the flow condition, outlet design, particle size, etc.) of microfluidic particle separation. The maximum Newtonian fluid flow rate in this study was set as 10 μL min^−1^; however, further increases in the throughput by acquiring *w*_n_ data for *Q*_n_ increase could be considered.

### Submicron separation of microspheres

**Fig. 6:**
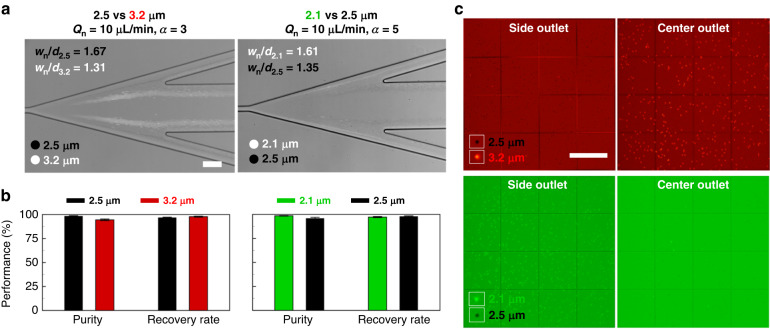
Submicron-resolution separation of microspheres. **a** The submicron-resolution microsphere separations of 2.5 vs 3.2 μm on the left and 2.1 vs 2.5 μm on the right side. The 2.1 and 3.2 μm fluorescent particles are tinged with white color, whereas the 2.5 μm mono particles have black color. Scale bar = 100 μm. **b** The efficiencies of submicron-resolution separation in terms of purity and recovery rate. **c** The hemocytometer images of microspheres collected from the side outlet and center outlet. Scale bar = 250 μm

The *w*_n_/*d* values according to flow conditions at *Q*_n_ = 10 μL min^−1^ are indicated for 2.1, 2.5, and 3.2 μm microspheres in Table 1. This table intuitively shows the regime of the microspheres’ equilibrium positions based on the analysis using the new dimensionless number. The 2.1-μm particle had *w*_n_/*d* = 1.61–3.19 under *α* = 1–5 flow conditions, indicating the inertial focusing regime (red) with an equilibrium position in the Newtonian fluid. The 2.5-μm microsphere at *α* = 5 had *w*_n_/*d* = 1.35, indicating the elastic focusing regime (sky blue). In other words, if the flow condition of *α* = 5 is selected for separating the 2.1-μm white fluorescent particle and 2.5-μm black mono particle at *Q*_n_ = 10 μL min^−1^, effective particle separation is possible, as shown in Fig. 6a (see Video S2, Supporting Information). Separation performance of 98.49% and 95.91% purity and 97.15% and 97.94% recovery rates, respectively, for each microsphere of 2.1 and 2.5 μm is shown in Fig. 6b. Similar to the separation of 2.1 and 2.5 μm particles, the separation conditions of 2.5 and 3.2 μm particles could be predicted by *w*_n_/*d* in Table 1. The flow condition of *α* = 3 could separate the 2.5-μm black mono particle and the 3.2-μm white fluorescent particle based on their inertial focusing regime and elastic focusing regime, respectively (see Video S3, Supporting Information). The 2.5 and 3.2 μm particles were separated with 98.41% and 94.50% purity and 96.87% and 97.74% recovery rates, respectively (Fig. 6b). The purity and recovery rate of submicron-resolution separation was calculated by the hemocytometer images of microspheres collected from the side outlet and center outlet (Fig. 6c). As the particle-to-particle size difference decreases, submicron-resolution separation occurred in a more limited range of the experimental conditions compared to the micro-resolution separation. However, the *w*_n_/*d* value could not only accurately predict the separation conditions of the submicron-resolution but also present *w*_n_ data for each *Q*_n_, this allowing increases in throughput despite the increased separation resolution compared with previous studies^29,31–33^.

**Table 1:**
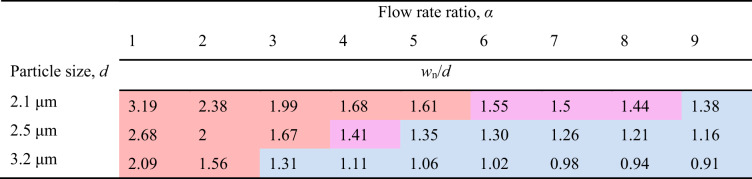
The values of *w*_n_/*d* under various *α* conditions at constant *Q*_n_ = 10 μL min^−1^ according to microsphere sizes 2.1, 2.5, and 3.2 μm

The red, purple, and sky blue cells indicate the inertial focusing reigme, elasto-inertial transition regime, and elastic focusing regime, respectively

### Separation of platelet from *Escherichia coli*

**Fig. 7:**
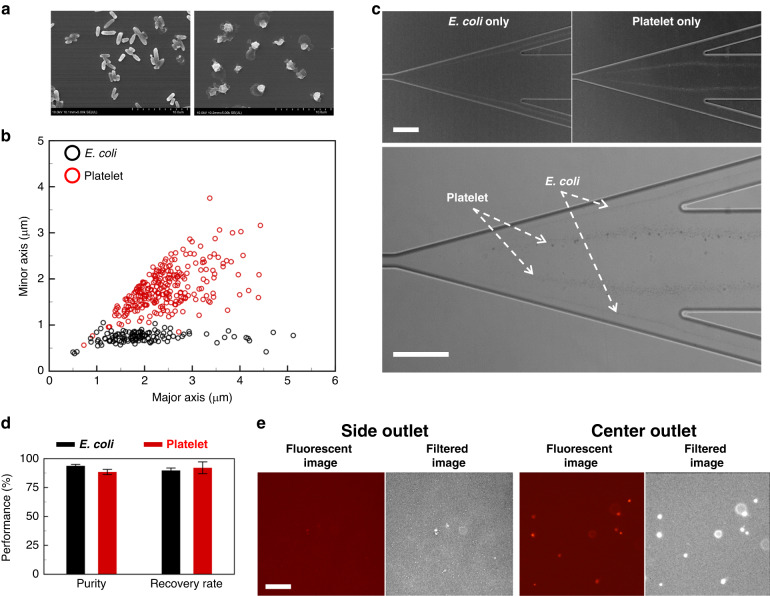
*Escherichia* coli and Platelet separaion. **a** Scanning electron microscope images of *E. coli* on the left side and platelet on the right side. **b** The length of major and minor axis of *E. coli* (black circle) and platelet (red circle). **c** The trajectories of *E. coli* and platelet under volumetric flow rate condition of *Q*_n_ = 10 μL min^−1^ and *Q*_ve_ = 50 μL min^−1^. *E. coli* and platelet move toward the side outlet and center outlet, respectively. Scale bar = 100 μm. **d** The efficiencies of *E. coli* and platelet separation in terms of purity and recovery rate. **e** The hemocytometer images of *E. coli* and platelet collected from the side outlet and center outlet. Scale bar = 50 μm

The separation of bio-particles with small-size, such as platelets and bacteria, is important to reduce the potential opportunity of disease/infection^41^. We have performed the elasto-inertial microfluidic separation of platelets from bacteria (*E. coli*), which has been regarded challenging due to their similar size^42^, as shown in Fig. 7a. The platelets were found to have the major axis = 2.44 ± 0.71 μm and minor axis = 1.79 ± 0.45 μm on average, while the *E. coli* was found to have the major axis = 2.06 ± 1.05 μm and minor axis = 0.74 ± 0.11 μm on average, as in Fig. 7b. The sample size of the two kinds of the biological samples was similar to the polymer microsphere separation experiments in Fig. 6a, where the 2.1 and 2.5 μm PS microspheres were used. Therefore, we applied the same flow conditions of *Q*_n_ = 10 μL/min and *α* = 5 for separation of the platelets from *E. coli*. We demonstrated the insignificance of the particle material in the proposed elasto-inertial microfluidic particle separation using the 3.2-μm polystyrene and 3.13-μm silica microspheres in Fig. S5 of Supporting Information. The experimental results in Fig. 7c show that the two types of the similar-sized bio-particles have distinct trajectories. The slightly larger-sized platelets migrated across the co-flow interface while the smaller bacteria remained in the Newtonian fluid (see Video S4, Supporting Information). The quantitative evaluation of the separation efficiency for the collected samples at the side and center outlets in Fig. 7d, e shows that the purity and recovery rate at the center outlet to target the platelets were 88.44 and 92.06%, respectively, while the purity and recovery rate were measured to be 93.74% and 89.75%, respectively, at the side outlets to target the bacteria. A slight decreased in the separation efficiency for the biological samples, compared to that for the polystyrene in Fig. 6, can be attributed to the size polydispersity of the biological samples, as in Fig. 7b.

## Conclusion

In this study, we propose a dimensionless analysis of elasto-inertial microfluidic separation of microspheres for precise prediction of the microsphere migration phenomenon across a co-flow of Newtonian and viscoelastic fluids. The proposed analysis is based on Reynolds number, modified Weissenberg number, modified elastic number, and newly introduced dimensionless number defined as the Newtonian fluid width divided by the microsphere diameter. Using these dimensionless numbers, we categorize the elasto-inertial microsphere lateral migration phenomenon into three regimes of inertial focusing, elasto-inertial transition, and elastic focusing based on particle equilibrium positions with reference to a co-flow interface of Newtonian and viscoelastic fluids. We experimentally validated the proposed analysis method with 2.1 and 3.2 μm PS microspheres under various flow conditions. We found that our estimation method can better characterize the microsphere lateral migration in elasto-inertial microfluidics. In addition to high-efficiency (>99%) separation of 2.1 and 3.2 μm microspheres, we further achieved the submicron-resolution separation of 2.1 and 2.5 μm, as well as 2.5 and 3.2 μm, PS microspheres at high throughput, purity, and recovery rate. For validation of practical applicability, we applied the proposed method for separation of similar-sized bio-particles: platelets from *E. coli*. We believe that the proposed dimensionless analysis can provide guidelines for the successful working conditions and estimation prior to experiments in the field of elasto-inertial microfluidic sample separation and purification.

## Experimental section

### Device fabrication

A master mold to fabricate Polydimethylsiloxane (PDMS) microchannels was prepared by a photolithography process using a negative photoresist (SU-2050, Kayaku, japan). The microchannel with a rectangular cross-section was fabricated by a soft lithography replica molding process. The PDMS base and curing agent (Sylard 184 A and 184B, Dow Corning, USA) were mixed in a ratio of 10:1 (w/w%), poured into the master mold, and left in an oven at 80 °C for 2 h. The PDMS stamp, in which the microchannel was patterned, was bonded with a slide glass by oxygen plasma bonding (Covance, Femto Science, Korea). The microfluidic chip was placed in an oven at 65 °C for 2 h to further enhance the bonding strength.

The main-microchannel was fabricated with a uniform height of 50 μm. The midstream microchannel, where the Newtonian and viscoelastic fluid co-flow is formed, was designed with a width of 20 μm. The channel length for the particles to have the equilibrium position in a rectangular channel was calculated by *L*_f_ = π*μh*^2^/*ρU*_m_*d*^2^*C*_li_—where *U*_m_ is the maximum flow velocity^37^—to be 11.87 mm at the slowest flow condition (*Q*_total_ = 2 μL min^−1^) and the smallest particle diameter (*d* = 2.1 μm) used in this experiment. The length of the midstream microchannel *L* was designed to be 15 mm so that all particles have the equilibrium position in the channel. In this study, we approximated that each particle has an equilibrium position under all flow conditions.

### Sample preparation

Two types of viscoelastic fluid with a concentration of 100 ppm were prepared by mixing PEO (*M*_w_ = 600 kDa, Sigma Aldrich, USA) powder in DI water for microsphere separation and 1× phosphate buffered saline (PBS) for bio-particle separation. The PEO solution was used after mixing with a magnetic stirrer for more than 24 h to ensure the complete dissolution of the PEO powder. Relaxation time of the 100 ppm PEO solution was obtained by the empirical formula *λ* = 18*λ*_Z_(*c*/c^*^)^0.65^ (ref. ^43^). Overlapping concentration is expressed as c^*^ = 0.77/[*μ*], and Zimm relaxation time *λ*_Z_ = *F*[*μ*]*M*_w_*μ*_s_/*N*_A_*k*_B_*T*—where *F* is the pre-factor 0.463, *μ*_s_ is the solution viscosity, *N*_A_ is Avogadro’s number, and *k*_B_ is Boltzmann’s constant—is determined according to Zimm’s theory^44,45^. The theoretical values of c^*^ and *λ*_Z_ can be obtained using the intrinsic viscosity [*μ*] = 0.072*M*_w_^0.65^ for the PEO solution according to the Mark–Houwink relationship^46^. The *λ* value of the 100-ppm PEO solution was calculated as 0.123 ms. We used the theoretical viscosity value of 100-ppm PEO solution for universal application of dimensionless analysis. The viscosity of PEO solution was calculated by 1.041 mPa∙s using polymer solution viscosity formula *μ* = *μ*_s_ + *μ*_p_ where *μ*_s_ is the solvent viscosity and *μ*_p_ = [*μ*]*cμ*_s_ is the polymeric contribution to the viscosity. The density of the synthesized PEO solution was measured using a density meter (DMA 35 Basic, Anton Paar, Austria).

The microsphere sample fluid was prepared by mixing PS particles with DI water. For particle trajectory analysis, the sample fluids were the mixture of *d* = 2.1 μm (green fluorescent particle, Thermo Fisher, USA) and 3.2 μm (red fluorescent particle, Thermo Fisher, USA) particles. Each sample used for submicron-resolution particle separation was mixed with *d* = 2.1 vs 2.5 μm (non-fluorescent particle, Thermo Fisher, USA) and 2.5 vs 3.2 μm particles. In all sample fluids, the concentration of each sized particle was 1 × 10^7^ particles mL^−1^, and the final particle concentration of the sample fluid was 2 × 10^7^ particles mL^−1^. For all microsphere sample fluids, Tween 20 (Sigma Aldrich, USA) was mixed at a concentration of 0.1 v/v% to prevent particle aggregation. Optionally, PEO coating inside the microchannel^47^ and device manufacture with copolymers PDMS-polyethylene glycol^48^ could be used to prevent adsorption of particles to the channel walls, but these methods were not used in this study due to the sufficient Re. The platelets were provided from the Korean Red Cross and stored in shaker-incubator (ES-20, Grant bio, UK) at 22 °C. Before the experiments, the platelets were dyed with red fluorescence by using an antibody labeling kit (Alexa Fluor^TM^ 568, Invitrogen, USA) and diluted 20 times with 1× PBS. The *E. coli* sample was cultured in a sterilized Luria-Bertani (LB) broth (L2542, Sigma Aldrich, USA) on a shaker-incubator at 37 °C for 24 h and diluted 10 times with 1× PBS.

### Flow visualization and measurements

The Newtonian/viscoelastic fluid interface was visualized by dispersing *d* = 300 nm (red fluorescent, Thermo Fisher, USA) PS particles in DI water at a concentration of 1 × 10^8^ particles mL^−1^. PS particles with *d* = 300 nm were injected in the direction of inlet 1 using a syringe pump (neMESYS Cetoni GmbH, Germany). Under the experimental conditions of this study, nanoparticles cannot migrate in the direction of the viscoelastic fluid owing to the *F*_el_ caused by the PEO solution. In addition, the Newtonian/viscoelastic fluid interface was measured at 100 μm near the upstream of the midstream microchannel. Therefore, the diffusion effect was not considered. Visualized images were precisely captured with a resolution of 0.0562 μm pixel^−1^ using an inverted microscope (IX73, Olympus, Japan), a CCD camera (E3ISPM, RisingCam, Japan).

Microsphere trajectories were observed at the downstream of the microchannel using an inverted microscope and captured via a high-speed camera (VEO 710 L, Phantom, USA). Images of the submicron-resolution separation were obtained by simultaneously using a halogen lamp and a mercury lamp, with fluorescent particles (2.1 and 3.2 μm) showing white color and non-fluorescent mono particles (2.5 μm) showing black color (Fig. 6a).

### Supplementary information

Supplementary Material

Supplementary Video S1

Supplementary Video S2

Supplementary Video S3

Supplementary Video S4
