# Supplementary material for: Elasto-inertial microfluidic separation of microspheres with submicron resolution at high-throughput
Source: Microsyst Nanoeng. 2024 Jan 22;10:15. doi: 10.1038/s41378-023-00633-w (PMC10803301; doi:10.1038/s41378-023-00633-w)
Supplement: Supplementary file 1 — Supplementary Material [file 41378_2023_633_MOESM1_ESM.docx]

Supporting Information

Elasto-Inertial Microfluidic Separation of Microspheres with Submicron Resolution at High-Throughput

Hyunwoo Jeon^1^, Song Ha Lee^1^, Jongho Shin^2^, Kicheol Song^2^, Nari Ahn^2^, and Jinsoo Park^1,^*

^1^Department of Mechanical Engineering, Chonnam National University, 77 Yongbong-ro Buk-gu, Gwangju, 61186, Republic of Korea

^2^Analytical Engineering Team, Samsung Display Co., Ltd., 181 Samsung-ro, Tangjeong-myeon, Asan-si, Chungcheongnam-do, 31454, Republic of Korea

* E-mail: jinsoopark@jnu.ac.kr

Fig. S1a shows that the 1 μm PS microspheres remained in the inertial focusing regime under varying flow conditions. Due to the small size, the w_n_/d value was larger than 1.55. In contrast, Fig. S1b shows that the 4 μm PS microspheres behaviors under varying flow conditions were in good agreement with those with 2.1 and 3.2 μm in Fig. 4.


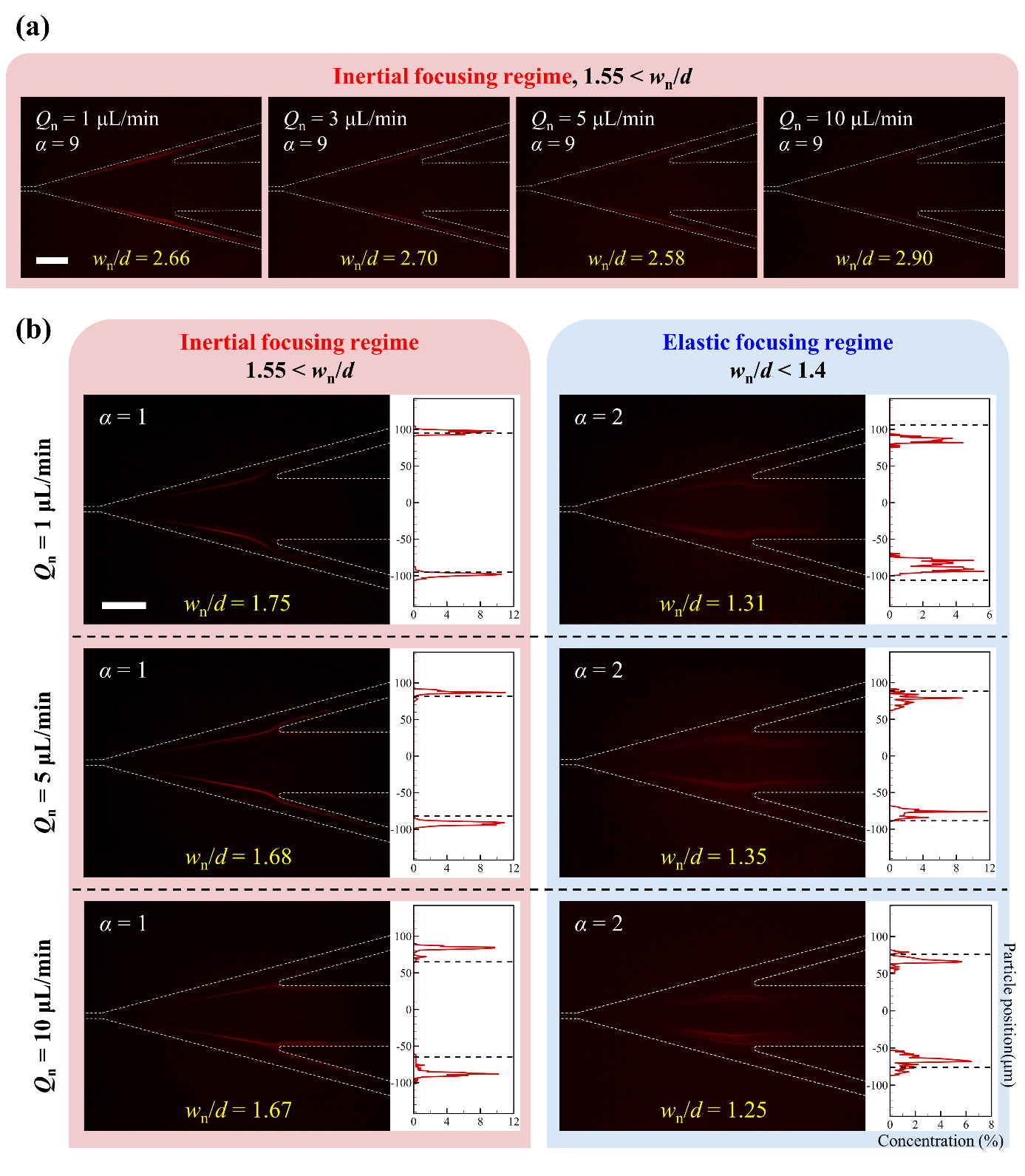


Fig. S1 Microsphere equilibrium positions according to relationship between particle size and Newtonian fluid width in the microchannel with *w* = 20 μm and *h* = 50 μm. Microspheres have different equilibrium positions under varying conditions of flow rate ratio according to Newtonian fluid flow rates. a *d* = 1 μm red fluorecent PS microsphere. Scale bar = 200 μm. b *d* = 4 μm red fluorecent PS microsphere. Scale bar = 200 μm.


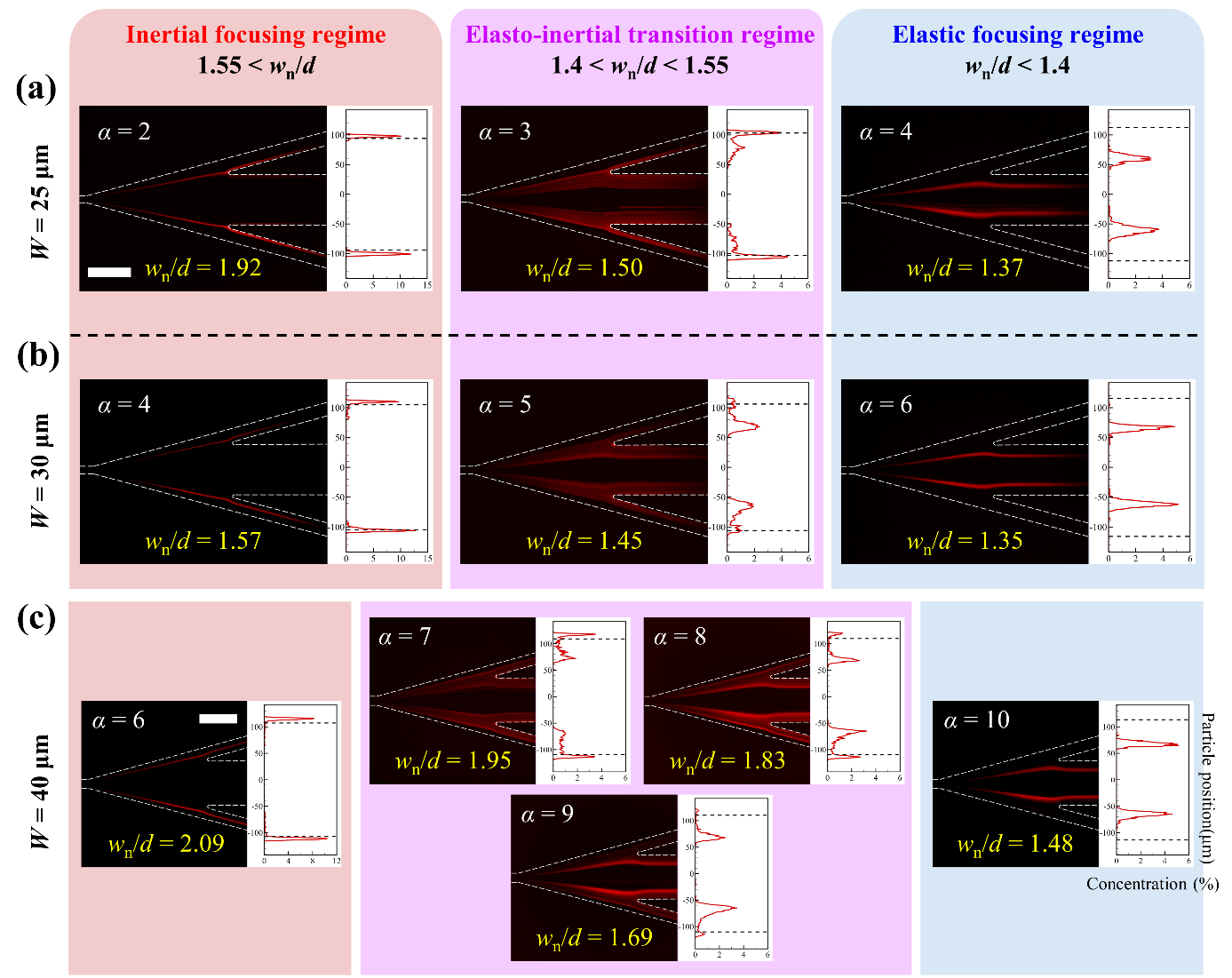
We investigated the trajectories of 3.2 μm red microspheres in Q_n_ = 10 μL min^−1^ under varying volumetric flow rate ratio (α) while the microchannel width was chaneged as w = 25, 30 and 40 μm and the microchannel height was fixed as h = 50 μm. In the case of w = 25 μm and 30 μm, Fig S2a, b show that the inertial focusing (1.55 < w_n_/d), elasto-inertial transition (1.4 < w_n_/d < 1.55), and elastic focusing (w_n_/d < 1.4) regimes were clearly observed, as in the case of w = 20 μm in Fig. 4. However, when the aspect ratio approached to 1 (close to square cross-section) with w = 40 μm and h = 50 μm, w_n_/d between the three regimes have been found to slightly increase, as in Fig S2c. These results could be due to decreasing the shear-gradient lift force that suppressed the migration of the particles to the Newtonian and viscoelastic fluids interface, as in Fig. 1c. In the high aspect microchannel, the shear-gradient lift force was sufficient in the y-direction (due to the steep velocity profile in the y-direction) anywhere away from the walls, but relatively weak in the z-direction^S1, S2^. In the square-like microchannel, the relatively blunt velocity profile in the y-direction reduced the shear-gradient lift force, and thus the microspheres moved toward the viscoelastic fluid at higher w_n_/d.

**Fig. S2** **The equilibrium positions of 3.2 μm microsphere according to relationship between particle size and Newtonian fluid width under varying microchannel width at fixed michannel height.** 3.2 μm microsphere have the different trajectories under varying *α* in fixed *Q*_n_ = 10 μL min^−1^ according to microchannel width **a** *w* = 25 μm, **b** *w* = 30 μm, and **c** *w* = 40 μm. Scale bar = 200 μm.

We have conducted the experiments with 6.02 μm polystyrene microspheres using a microchannel with w = 40 μm and h = 100 μm in Fig. S4. Even with the larger particles and extended microchannel, our dimensionless analysis to estimate the particle migration behavior in elasto-inertial microfluidics was found to be valid. The experimental results show that the inertial focusing (1.55 < w_n_/d), elasto-inertial transition (1.4 < w_n_/d < 1.55), and elastic focusing (w_n_/d < 1.4) regimes were clearly observed, as in the results with the 2.1 and 3.2 μm microspheres in Fig. 4.


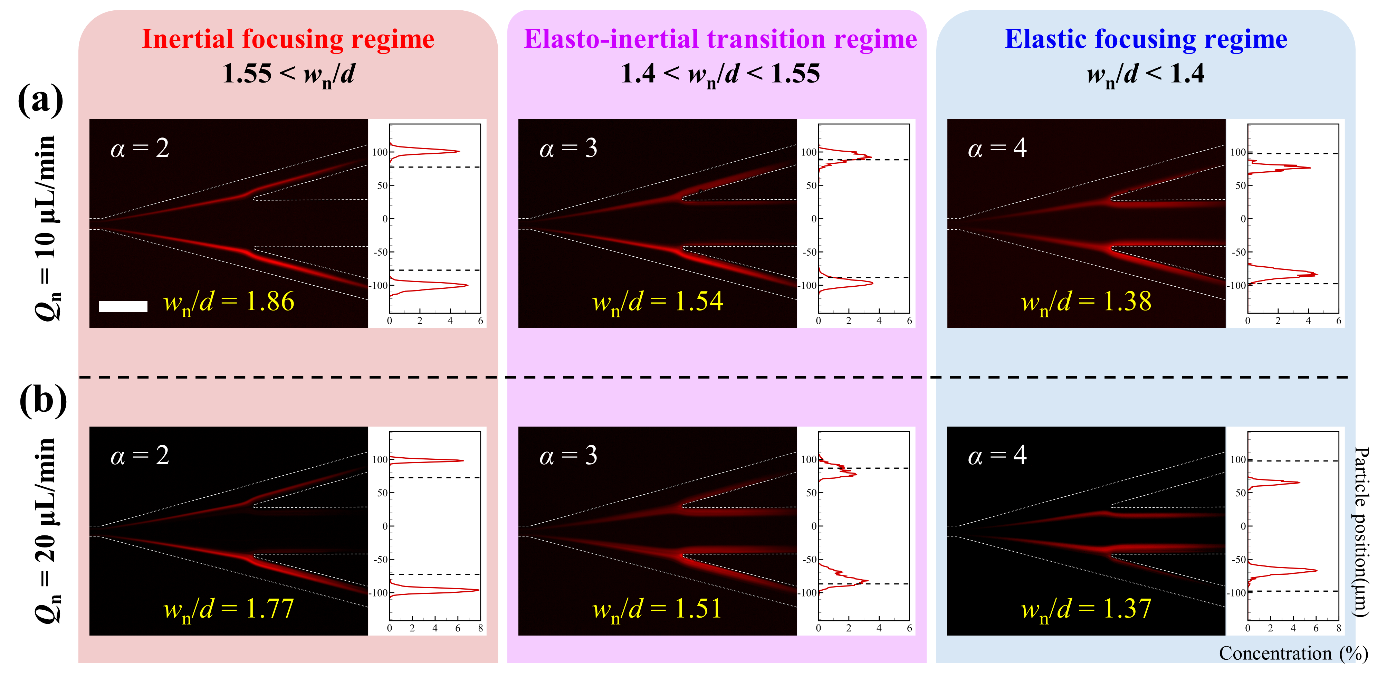


Fig. S3 The equilibrium positions of 6.02 μm microsphere according to relationship between particle size and Newtonian fluid width in the microchannel of *w* = 40 μm, *h* = 100 μm and *L* = 30 mm. a *Q*_n_ = 10 μL min^−1^ and b *Q*_n_ = 20 μL min^−1^. Scale bar = 200 μm


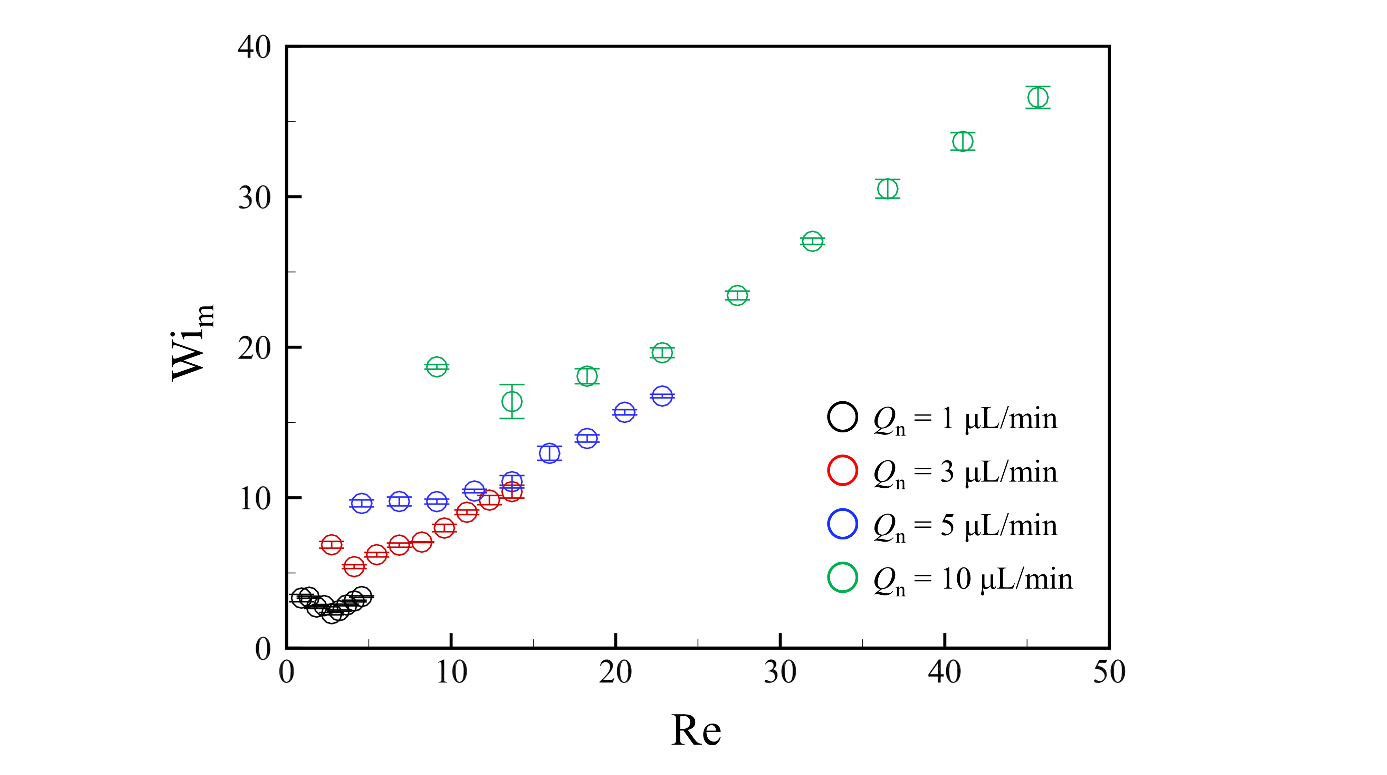


Fig. S4 The diagram of Reynolds number and modified weissenberg number in the various experimental conditions of in this study. The black, red, blue, and green circles indicate the volumetric flow rate condition of Newtonian fluid 1, 3, 5, and 10 μL min^−1^, respectively, in various *α* = 1–9.

Fig. S4 shows all experimental conditions, which are expressed by the Reynolds number and modified Weissenberg number. Wi_m_ is a function of the viscoelastic fluid width and the viscoelastic fluid flow rate when the flow condition changes in the same viscoelastic fluid properties. In the low α regime, the rapid interface change of co-flow described in Fig. 2b led to a slight decrease in Wi_m_, and as α and Re increased, flow rate change became dominant in Wi_m_ because the change of interface position was insignificant. Fig. S4 could be analyzed from the viewpoint of particle behavior using the changes Re, Wi_m_, and El_m_ (Wi_m_/Re) in different α conditions of fixed volumetric flow rate of Newtonian fluid, however, it is difficult to understand a correlation in all flow conditions and consistent interpretation of particle behavior.

**
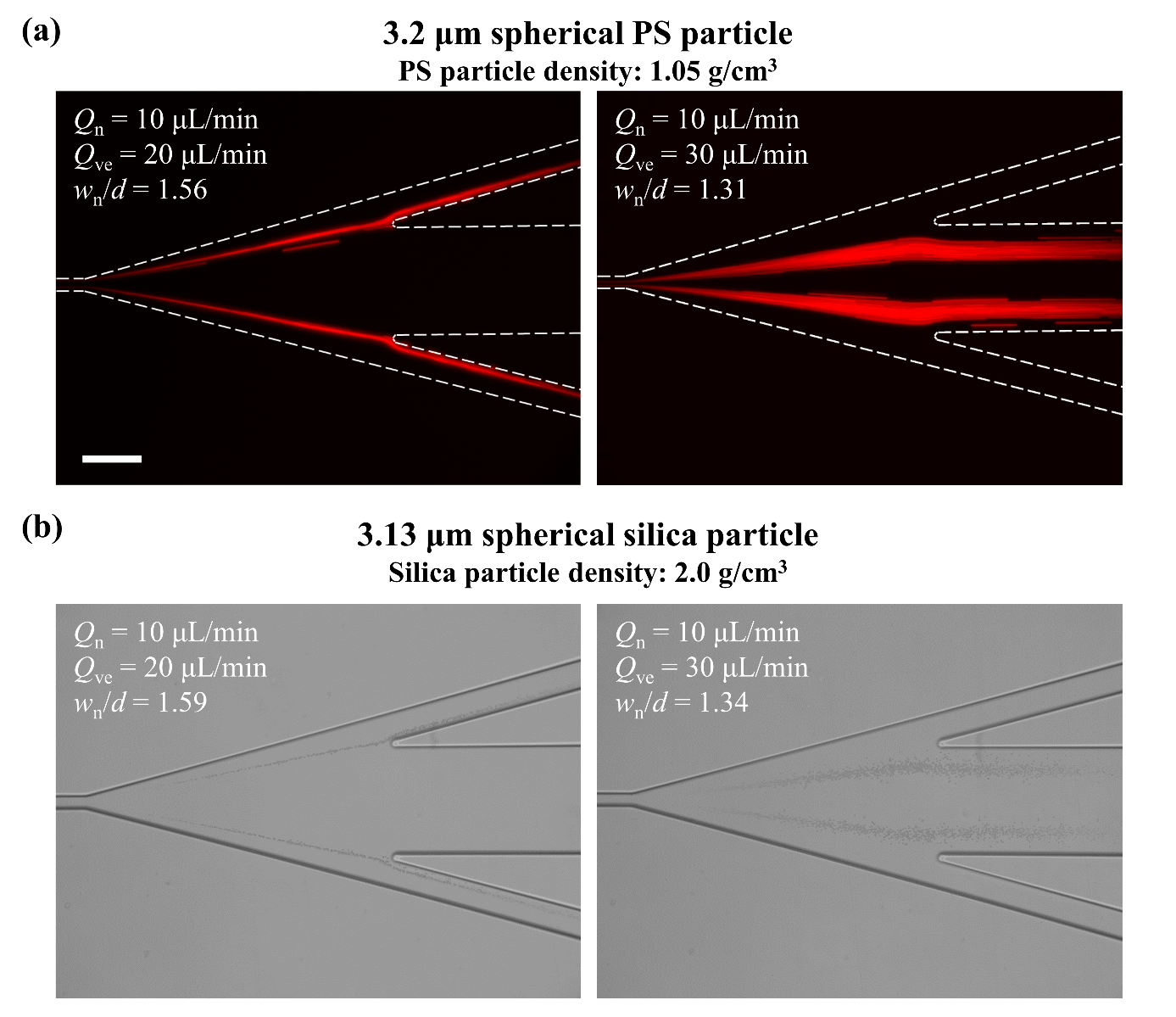
**We have conducted the experiments to demonstrate the insignificance of the particle material in the proposed elasto-inertial microfluidic microsphere separation using the 3.2 μm polystyrene and 3.13 μm silica microspheres in Fig. S5. Under the same flow rate conditions, the two types of the microspheres showed the same trajectory by the elasto-inertial migration. With Q_n_ = 10 μL min^−1^ and Q_ve_ = 20 μL min^−1^, the wn/d value was 1.56 and 1.59 for polystyrene and silica, respectively. The slight difference in wn/d was attributed to the slight size difference. Both types of the particles remained close to the microchannel side walls because the inertial effect was less dominant than the elastic effect in the inertial focusing regime. In contrast, the w_n_/d value was 1.31 and 1.34 for polystyrene and silica, respectively, with Q_n_ = 10 μL min^−1^ and Q_ve_ = 30 μL min^−1^. In the elastic focusing regime, both microspheres migrate across the Newtonian-viscoelastic fluids interface due to the dominant inertial effect compared to the elastic effect at the co-flow interface. The insignificance of the material properties allows more wide and practical applications of the proposed elasto-inertial microsphere separation method.

Fig. S5 The particle trajectories according to different material types. a polystyrene particle with density = 1.05 g/cm^3^ and b sillca particle with density = 2.0 g/cm^3^

**Table S1** **The particle sizes and separation conditions of elasto-inertial microfluidics used in reference studies. This table indicates why reference studies selected or avoided the corresponding separation condition according to *w*_n_/*d*.**

| Reference | Object | Flow rate | *d* | *w*_n_/*d* | Regime |
| --- | --- | --- | --- | --- | --- |
| Tian et *al*. (2017)^S1^ | Polystyrene | *Q*_n_ = 5 μL min^−1^  *Q*_ve_ = 40 μL min^−1^ | 1 μm | 2.77 | Inertial focusing |
|  |  |  | 2 μm | 1.38 | Elastic focusing |
| Liu et *al*. (2020)^S2^ | S. Cerevisiae | *Q*_n_ = 1 μL min^−1^  *Q*_ve_ = 5 μL min^−1^ | 2.8 μm | 1.18 | Elastic focusing |
|  |  |  | 5.05 μm | 0.65 | Elastic focusing |
|  |  |  | 7.70 μm | 0.43 | Elastic focusing |
| Zhang et *al*. (2023)^S3^ | Polystyrene | *Q*_n_ = 1 μL min^−1^  *Q*_ve_ = 9 μL min^−1^ | 1 μm | 2.66 | Inertial focusing |
|  |  |  | 4.8 μm | 0.56 | Elastic focusing |

Tian et *al*. (2017) selected flow conditions of *Q*_n_ = 5 and *Q*_ve_ = 40 μL min^−1^ for separating 1 and 2 μm polystyrene particles^S3^. We could predict that the reason why the separation condition was selected was that *w*_n_/*d* values of 1 and 2 μm particles indicate the regime of inertial focusing and elastic focusing while having values of 2.77 and 1.38, respectively. In the same way, the separation condition of Zhang et *al*. (2023) was selected for separating 1 and 4.8 μm particles^S5^. Liu et *al*. (2020) selected the 1000 ppm PEO solution (*M*_w_ = 600 kDa) for separating the Saccharomyces cerevisiae, compared to other studies selecting the 100 ppm PEO solution^S4^. All particles having effective diameters of 2.8, 5.05, and 7.70 μm belonged in the elastic focusing regime due to the low elastic lift force acting toward the microchannel walls. Thus, they had to choose the 1000 ppm PEO solution that could enhance the elastic lift force to separate the particles.

**Video S1**. The supplementary video of Fig. 5b. The separation of 2.1 (green) and 3.2 μm (red) fluorescent microspheres using the new dimensionless analysis *w*_n_/*d*. The 2.1 and 3.2 μm microspheres had *w*_n_/*d* = 1.68 (inertial focusing regime) and 1.11 (elastic focusing regime), respectively, at specific flow rate conditions *Q*_n_ = 10 μL min^−1^ and *Q*_ve_ = 40 μL min^−1^.

**Video S2**. The supplementary video of Fig 6a. The submicron-resolution microsphere separations of 2.5 (black) and 3.2 μm (white) using the new dimensionless analysis *w*_n_/*d*. The 2.5 and 3.2 μm microspheres had *w*_n_/*d* = 1.67 (inertial focusing regime) and 1.30 (elastic focusing regime), respectively, at specific flow rate conditions *Q*_n_ = 10 μL min^−1^ and *Q*_ve_ = 30 μL min^−1^.

**Video S3**. The supplementary video of Fig. 6a. The submicron-resolution microsphere separations of 2.1 (white) and 2.5 μm (black) using the new dimensionless analysis *w*_n_/*d*. The 2.1 and 2.5 μm microspheres had *w*_n_/*d* = 1.61 (inertial focusing regime) and 1.35 (elastic focusing regime), respectively, at specific flow rate conditions *Q*_n_ = 10 μL min^−1^ and *Q*_ve_ = 50 μL min^−1^.

**Video S4**. The supplementary video of Fig. 7c. The bio-particles separation of *E. coli* and platelet at specific flow rate conditions *Q*_n_ = 10 μL min^−1^ and *Q*_ve_ = 50 μL min^−1^.

**References**

S1. Amini, H., Lee, W. & Di Carlo, D. Inertial microfluidic physics. Lab Chip 14, 2739-2761 (2014).

S2. Zhou, J. & Papautsky, I. Fundamentals of inertial focusing in microchannels. Lab Chip 13, 1121-1132 (2013).

S3. Tian, F. et al. Microfluidic co-flow of Newtonian and viscoelastic fluids for high-resolution separation of microparticles. Lab Chip 17, 3078-3085 (2017).

S4. Liu, P. et al. Separation and Enrichment of Yeast Saccharomyces cerevisiae by Shape Using Viscoelastic Microfluidics. Anal. Chem. 93, 1586-1595 (2021).

S5. Zhang, T. et al. Microfluidic Separation and Enrichment of Escherichia coli by Size Using Viscoelastic Flows. Anal. Chem. 95, 2561-2569 (2023)
